# Supplementary material for: Constructing synthetic nuclear architectures via transcriptional condensates in a DNA protonucleus
Source: Nat Commun. 2025 Sep 10;16:8254. doi: 10.1038/s41467-025-63445-8 (PMC12423304; doi:10.1038/s41467-025-63445-8)
Supplement: Supplementary file 1 — Supplementary Information [file 41467_2025_63445_MOESM1_ESM.pdf]

Supplementary Information

Constructing synthetic nuclear architectures via transcriptional condensates in a DNA protonucleus

Miao Xie[1,2,#]*, Weixiang Chen[1,2,#], Maria Vonk-de Roy[1], Andreas Walther[1,2]*

Affiliations

[1]Life-Like Materials and Systems, University of Mainz, Duesbergweg 10-14, 55128 Mainz, Germany.

[2]Max Planck Institute for Polymer Research, 55128 Mainz, Germany.

*Corresponding author. Email: miao.xie@uni-mainz.de; andreas.walther@uni-mainz.de

[#]These authors contributed equally.

Supplementary Figure 1. Preparation of PN containing promoter sequences (p) inside................................3

Supplementary Figure 2. ssDNA template hinders the formation of transcriptional KL condensates in both solution and PN...4

Supplementary Figure 3. Formation of transcriptional KL1 condensates in solution at different NTP concentrations. 5

Supplementary Figure 4. Formation of single transcriptional KL1 condensates in PN. 6

Supplementary Figure 5. Half-bleaching experiment on KL1 condensates in solution and in PN. 7

Supplementary Figure 6. Pre-equilibrium of T7 RNAP enables a more homogenous transcription inside PN. 8

Supplementary Figure 7. Design and transcription of KL1-BrA in solution and in PN. 9

Supplementary Figure 8. Formation of transcriptional KL1-PN co-condensate deposited at the bottom of host PN...10

Supplementary Figure 9. Dynamic properties of KL1 phase and PN matrix phase in the KL1-PN co-condensate quantified by FRAP. 11

Supplementary Figure 10. NUPACK simulation showing the absence of specific interaction between A_{20}-o ssDNA and KL1 sequence. 12

Supplementary Figure 11. Invasion of o*-Atto647 at 100 mM Mg^{2+} shows slower disassembly of KL-PN co-condensate compared to 30 mM Mg^{2+}. 13

Supplementary Figure 12. Formation of KL1-R1 and KL2-R2 condensates in PN versus KL2-R2 transcribed in solution and recruited into PN. 14

Supplementary Figure 13. Transcription kinetics of KL1-R1 and KL2-R2 in solution. 15

Supplementary Figure 14. Effect of asymmetric template concentrations for orthogonal KLs on the formation of condensates in PN...16

Supplementary Figure 15. Preferred partitioning of A_{20}-o ssDNA into KL1-R1 condensates and exclusion of A_{20}-o/o* dsDNA from both KL1-R1 and KL2-R2 condensates. 17

Supplementary Figure 16. Binding interaction between A_{20}-o, KL1-R1, and KL2-R2. 18

Supplementary Table 1. Oligomers for PN and labels, with their names, sequences, purification methods, modifications, and suppliers. 19

Supplementary Table 2. Oligomers for kissing loop condensate transcription, with their names, sequences, purification methods, modifications, and suppliers. 21

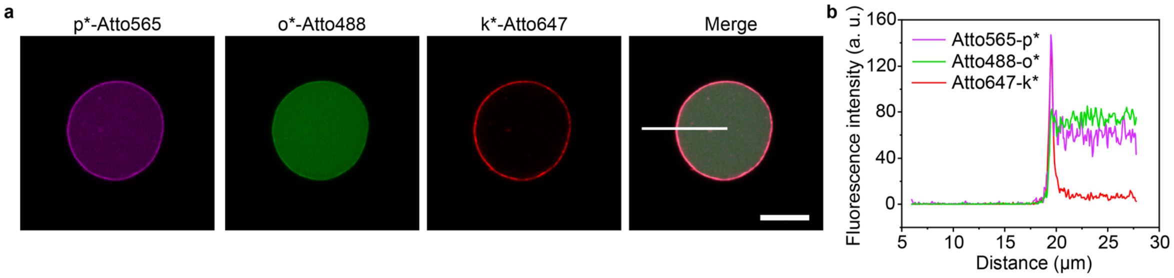

Supplementary Figure 1. Preparation of PN containing promoter sequences (p) inside.

a, Representative CLSM images of the PN containing different core (p and o) and shell (k) barcodes, labeled by their complementary ssDNA strands (k*-Atto647, p*-Atto565, and o*-Atto488). **b**, Fluorescence intensity profiles corresponding to the line segment analysis along the white line in (**a**) in three channels, showing a core-shell structure with poly(A_{20}-p)$_n$ and poly(A_{20}-o)$_n$ colocalized at the interior of the PN, and poly(T_{20}-k)$_n$ at the PN shell. Scale bar: 10 μm.

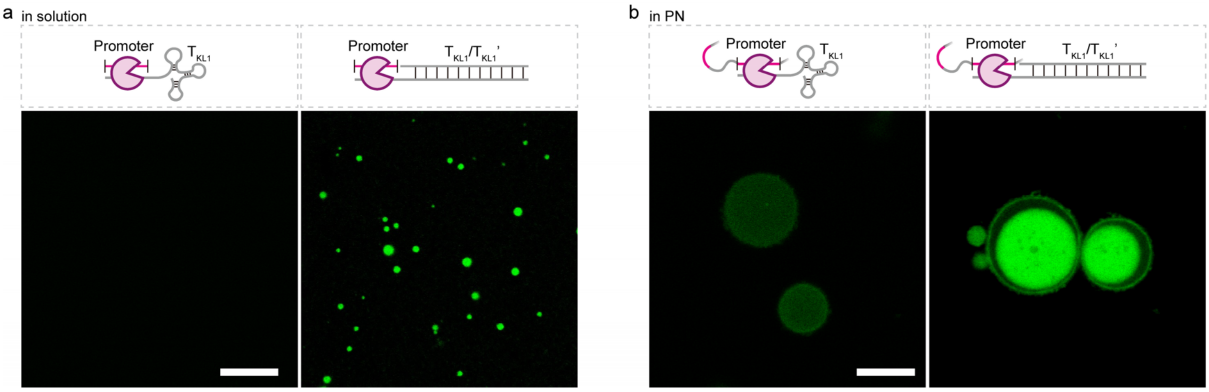

Supplementary Figure 2. ssDNA template hinders the formation of transcriptional KL condensates in both solution and PN.

a, Representative CLSM images of transcriptional KL condensate in solution with [NTP] : [T_{KL1}] = 7.2, transcribed by promoter ssDNA, which is hybridized with T_{KL1} as a ssDNA template or T_{KL1}/T_{KL1}' as a dsDNA template ([T_{KL1}] : [p] = 1 : 1, 30 °C, 30 mM Mg^{2+}, 2.5 U/µL T7 RNAP). Note that [NTP] is set to a concentration where condensation in solution appears (see Supplementary Figure 3). **b,** Representative CLSM images of transcriptional KL in PN with ssDNA template or dsDNA template for transcription ([NTP] : [T_{KL1}] = 3.6, 30 °C, 30 mM Mg^{2+}, 2.5 U/µL T7 RNAP). Scale bar: 10 µm.

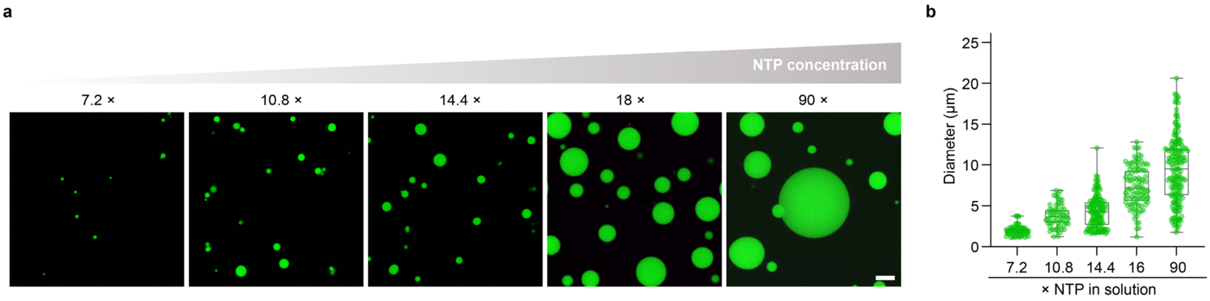

Supplementary Figure 3. Formation of transcriptional KL1 condensates in solution at different NTP concentrations.

a, Representative CLSM images of in-solution transcriptional KL1 condensates at various [NTP] : [T_{KL1}] ratios from 7.2 to 90, transcribed by promoter ssDNA ([T_{KL1}] : [p] = 1 : 1). **b**, Diameter distribution at different NTP concentrations. Box plot (**b**): median (central line), interquartile range (box), min-max (whiskers). N = 61 – 129 from 3 independent experiments. Scale bar: 10 µm.

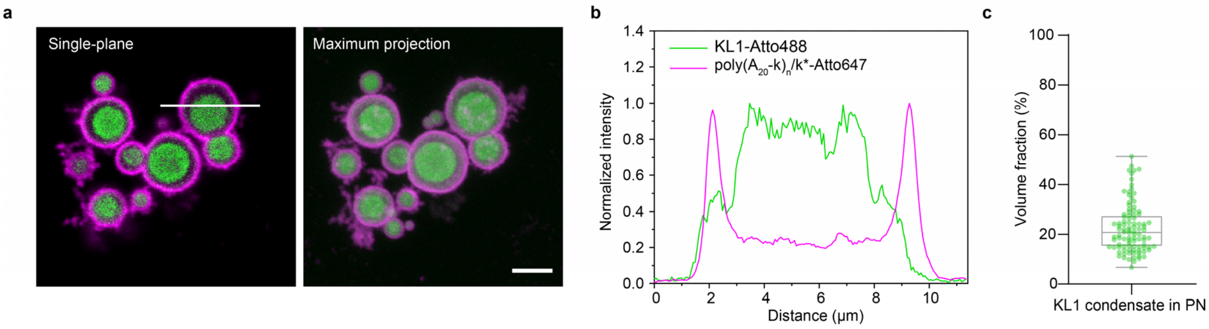

Supplementary Figure 4. Formation of single transcriptional KL1 condensates in PN.

a, Representative single-plane CLSM image and maximum intensity projection of z-stacked CLSM images showing the formation of single KL1 condensates in each PN ([NTP] : [T_{KL1}] = 3.6 : 1, 30 °C, 30 mM Mg^{2+}, 2.5 U/μL T7 RNAP, 18 h reaction). Green channel: KL1 condensate (labeled by UTP-Atto488); Magenta channel: PN shell (labeled by k*-Atto647). Scale bar: 5 μm. **b**, Normalized intensity profiles corresponding to the line segment analysis along the white line in (**a**) showing the distribution of KL1 condensates with a spongy structure, and the PN shell. **c**, Volume fraction of single transcriptional KL1 condensate relative to the host PN. Box plot (**c**): median (central line), interquartile range (box), min-max (whiskers). $N = 98$ from 3 independent experiments. This figure relates to Fig. 2e in the main text.

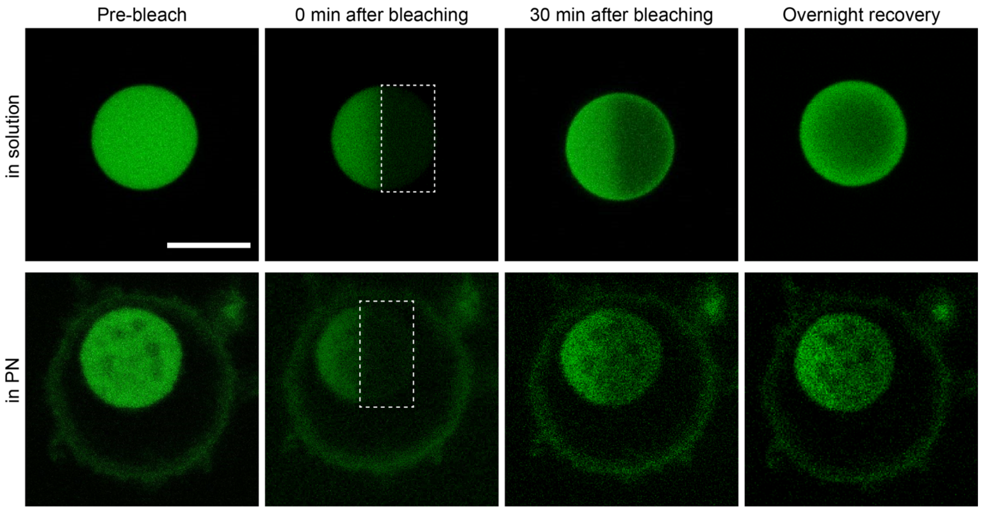

Supplementary Figure 5. Half-bleaching experiment on KL1 condensates in solution and in PN. Representative CLSM images showing the transcriptional KL1 condensates in solution and in PN, before and after bleaching at different times. The bleached regions are indicated by the white dashed rectangle. Scale bar: 5 μm.

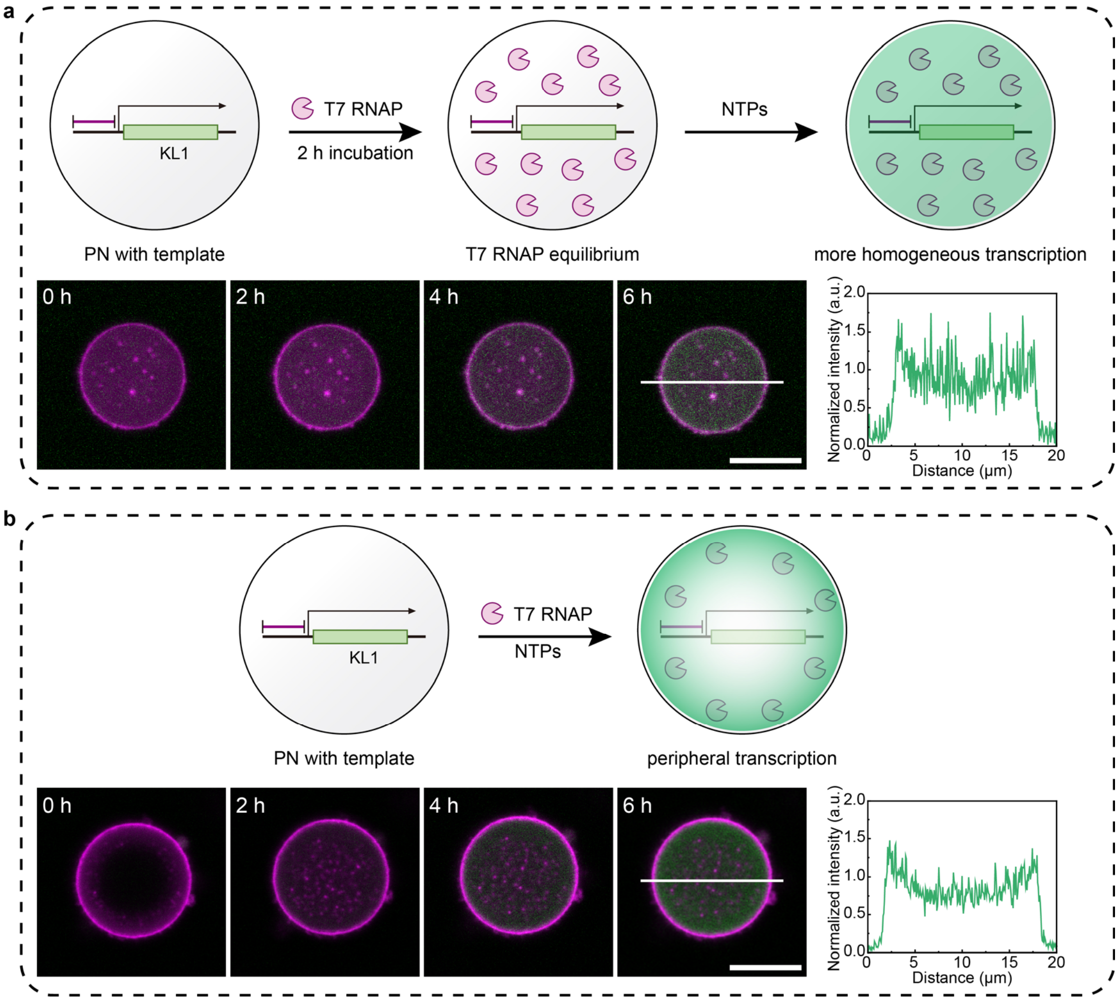

Supplementary Figure 6. Pre-equilibrium of T7 RNAP enables a more homogenous transcription inside PN.

Rationale: To investigate if the diffusion of T7 RNAP into PN has an impact on the peripheral transcription, we performed transcription of KL1 inside PN equilibrated with T7 RNAP prior the addition of NTPs. **a**, scheme and representative CLSM images of KL1 transcription in PN pre-equilibrated with T7 RNAP for 2 h, before the addition of NTPs to trigger transcription. The plot shows cross-sectional line profile along the white line in the CLSM image at 6 h, demonstrating a more homogenous transcription inside the PN. **b**, scheme and representative CLSM images of KL1 transcription in PN with simultaneous addition of T7 RNAP and NTPs (standard conditions used in the main text). The plot shows cross-sectional line profile along the white line in the CLSM image at 6 h, demonstrating a non-homogenous and peripheral transcription inside the PN. Green channel: KL1 condensate labeled by UTP-Atto488; Magenta channel: PN shell (poly(T_{20}-k)$_n$ labeled with k*-Atto647). [NTP] : [T_{KL1}] = 3.6 : 1, 1 mol% UTP-Atto488, 30 °C, 2.5 U/μL T7 RNAP, 30 mM Mg^{2+}. Note that we only focus on the first 6h of co-transcriptional condensation, because afterwards it's rather the maturation of the structure/co-condensate. Scale bars: 10 μm.

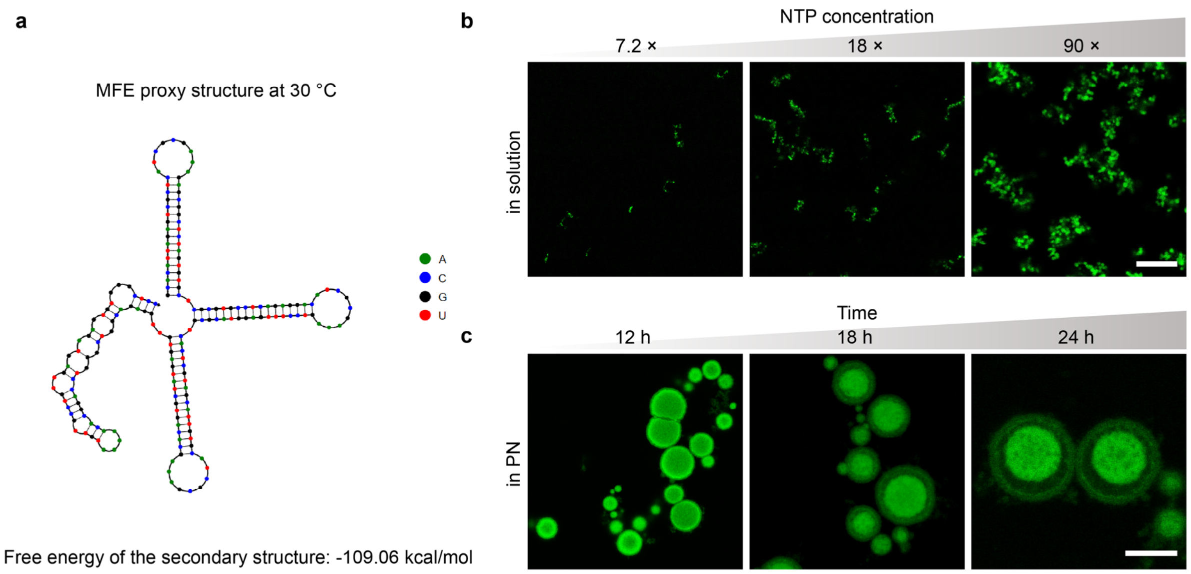

Supplementary Figure 7. Design and transcription of KL1-BrA in solution and in PN.

a, NUPACK-simulated structure of KL1-BrA at 30 °C. **b**, Transcription and assembly of KL1-BrA in solution at varying [NTP] : [T_{KL1}] ratios from 7.2 to 90 ([T_{KL1}] : [p] = 1 : 1, 30 °C, 30 mM Mg^{2+}, 24 h reaction). **c**, Formation of transcriptional KL1-BrA condensates in PN over 12-24 h ([NTP] : [T_{KL1}] : [p] = 3.6: 1 : 1, 30 °C, 30 mM Mg^{2+}, 2.5 U/μL T7 RNAP). Scale bars: 10 μm.

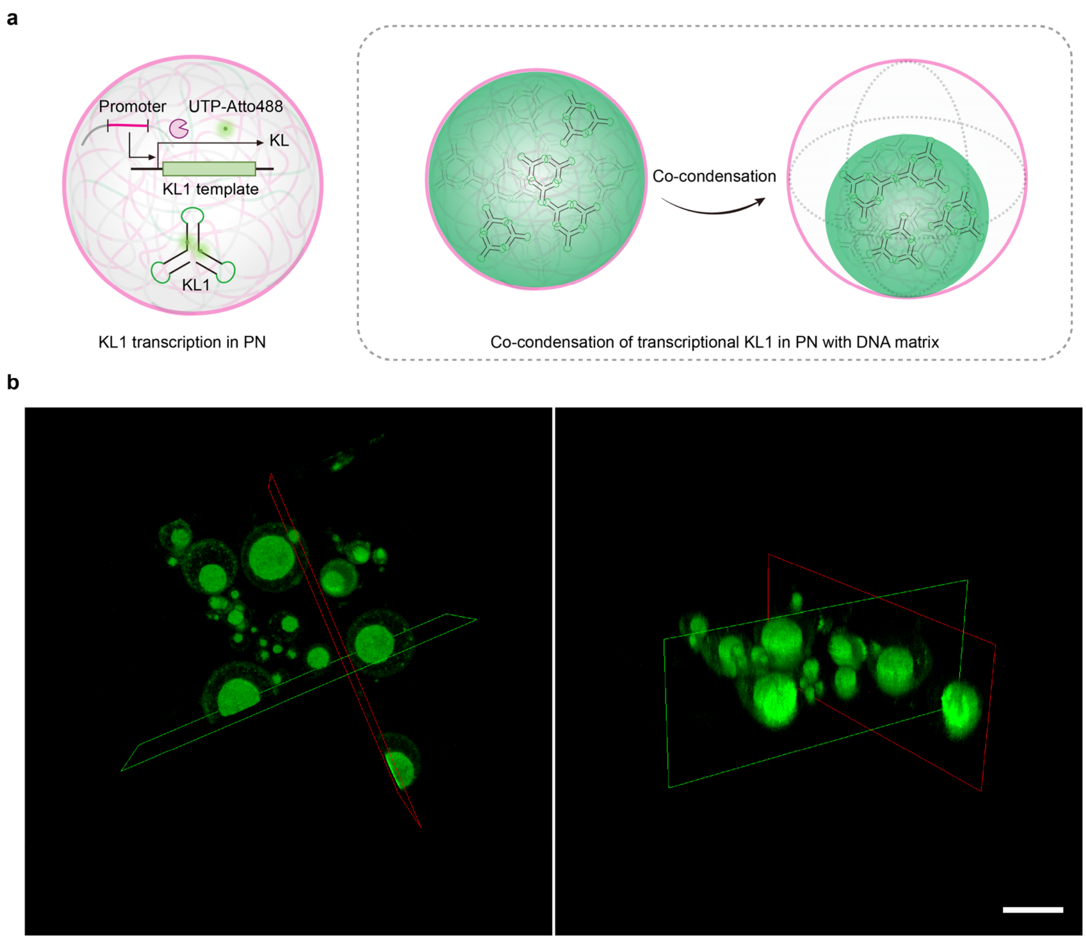

Supplementary Figure 8. Formation of transcriptional KL1-PN co-condensate deposited at the bottom of host PN.

a, Schematic illustration of KL1-PN co-condensation between KL1 transcripts and DNA matrix of the host PN induced by localized transcription of KL1 in the PN. **b**, Representative 3D CLSM images showing the top (left) and front (right) views of co-condensates in PN. Scale bar: 10 μm.

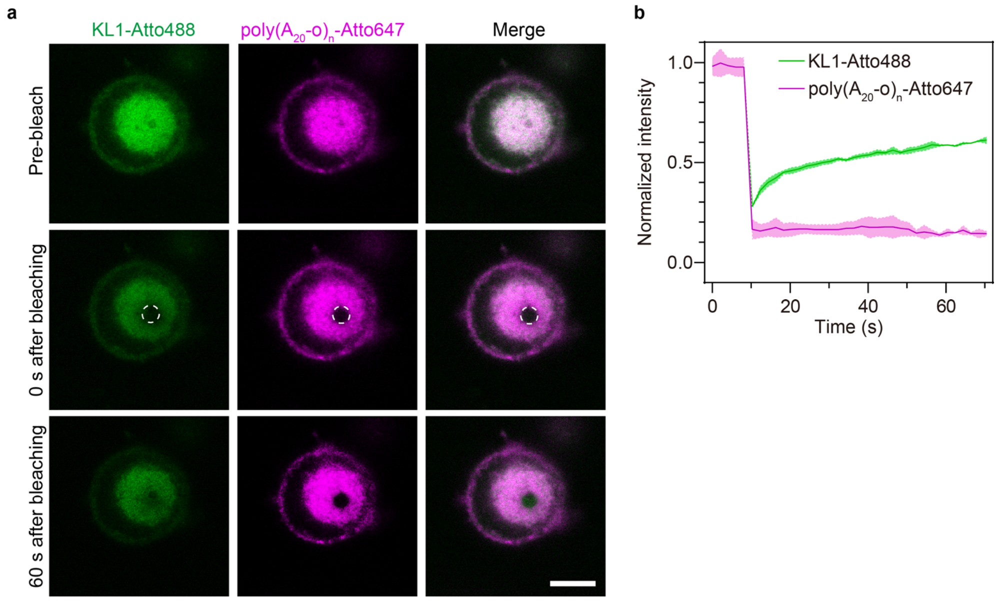

Supplementary Figure 9. Dynamic properties of KL1 phase and PN matrix phase in the KL1-PN co-condensate quantified by FRAP.

a, Representative CLSM images showing KL1 condensate phase (green channel) and PN matrix phase (magenta channel) of a co-condensate in PN before and after bleaching at 30 °C. The white dashed circles indicate the bleached regions. **b**, Normalized fluorescence recovery kinetics in the bleached regions in (**a**) for the KL1 phase and the PN matrix phase, quantifying the molecular diffusivity of co-condensates in PN at 30 °C. Intensity values were normalized to pre-bleached levels. Data are presented as mean ± SD ($N = 4$ independent experiments). Scale bar: 5 µm.

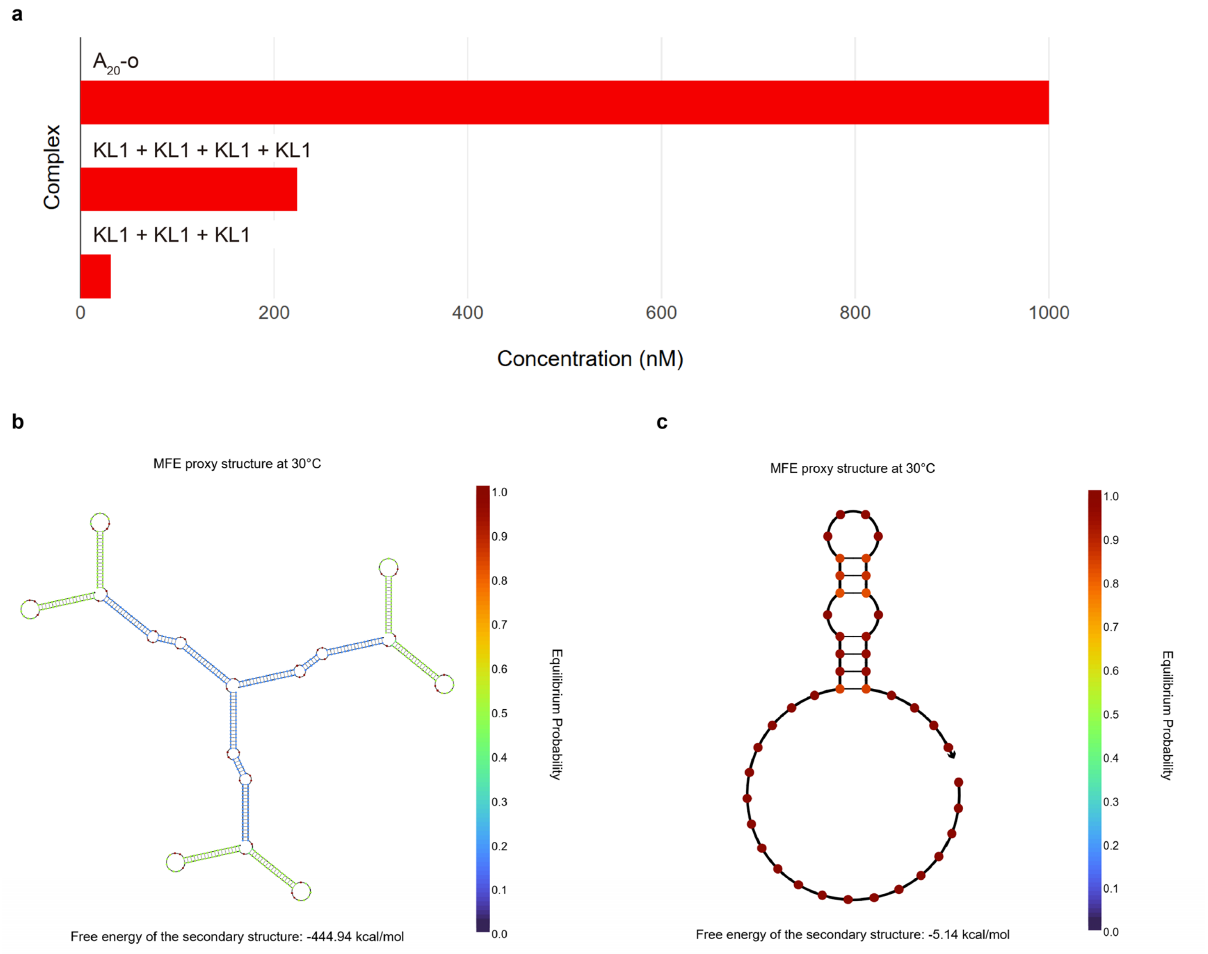

Supplementary Figure 10. NUPACK simulation showing the absence of specific interaction between A_{20}-o ssDNA and KL1 sequence.

a, Simulated complex structures formed by 1 μM A_{20}-o (ssDNA) and 1 μM KL1 (ssDNA) at 30 °C. **b**, NUPACK-simulated structures of KL1+ KL1+ KL1+ KL1. **c**, NUPACK-simulated structures of A_{20}-o. No complex structure between A_{20}-o and KL1 is formed. Note that all simulations are based on DNA sequences.

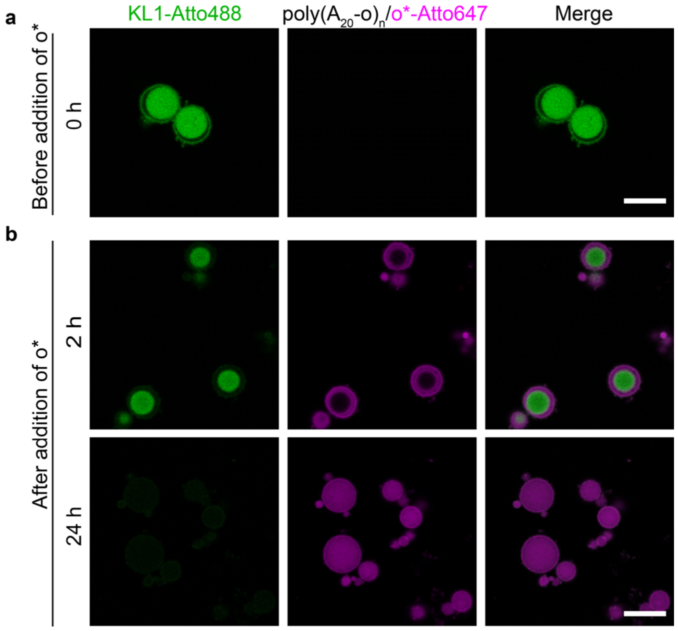

Supplementary Figure 11. Invasion of o*-Atto647 at 100 mM Mg^{2+} shows slower disassembly of KL-PN co-condensate compared to 30 mM Mg^{2+}.

a, Representative CLSM images of KL-PN co-condensate formed by KL1 transcription in PN with 30 mM Mg^{2+} (KL1-Atto488, green channel). The overall Mg^{2+} concentration was then increased to 100 mM, and the solution was incubated for 24 h before the addition of o*-Atto647 to hybridize with poly(A_{20}-o)$_n$ in PN (magenta channel). **b,** Representative CLSM images of KL-PN co-condensate at 2 or 24 h after o*-Atto647 addition at 100 mM Mg^{2+}. Scale bar: 10 µm.

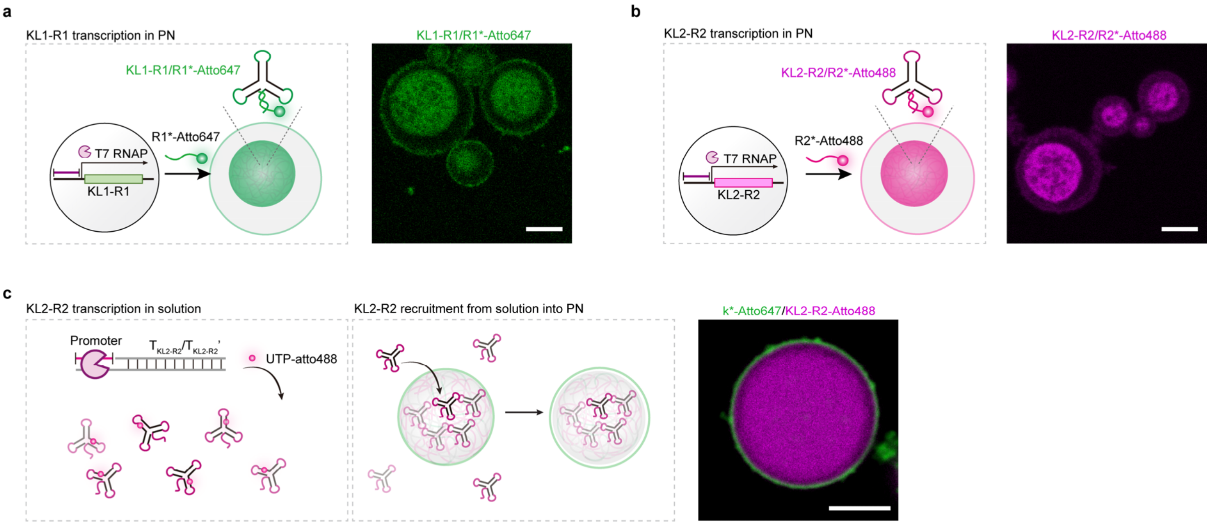

Supplementary Figure 12. Formation of KL1-R1 and KL2-R2 condensates in PN versus KL2-R2 transcribed in solution and recruited into PN.

a, Scheme and representative CLSM image showing the formation of single condensates in PN by localized transcription of KL1-R1. The condensate is labeled by R1*-Atto647 (green channel). **b**, Scheme and representative CLSM image showing the formation of single condensates in PN by localized transcription of KL2-R2. The condensate is labeled by R2*-Atto488 (magenta channel). 2.5 U/µL T7 RNAP, 30 mM Mg^{2+}, 30 °C, [NTP] : [R1* or R2*] : [T_{KL1-R1} or T_{KL1-R1}] : [p] = 3.6 : 3.6 : 1 : 1, 18 h reaction for both (**a**) and (**b**). **c**, Scheme and representative CLSM images for the transcription of KL2-R2 in solution, which gets recruited into the PN. KL2-R2 was first transcribed in solution with UTP-Atto488 feeding ([NTP] : [T_{KL2-R2}] = 3.6 : 1, 1 mol% UTP-Atto488, 30 °C, 2.5 U/µL T7 RNAP, 30 mM Mg^{2+}). Pristine PN was added and incubated with the existing KL2-R2 for 18 h for recruitment at 30 °C with 30 mM Mg^{2+}. Green channel: PN shell labeled with k*-Atto647; Magenta channel: KL2-R2-Atto488. Scale bars: 5 µm (**a**, **b**); 10 µm (**c**).

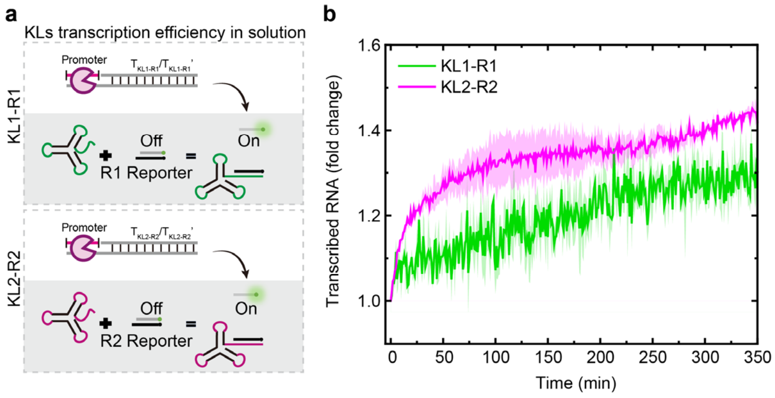

Supplementary Figure 13. Transcription kinetics of KL1-R1 and KL2-R2 in solution.

a, Scheme showing transcription of KL1-R1 (top) or KL2-R2 (down) in solution characterized by plate reader. To quantify the transcribed KLs RNA, dsDNA reporters with fluorophore-quencher pairs are present in solutions to react with dangling strand of transcribed KLs (R1 Reporter for KL1-R1, R2 Reporter for KL2-R2) by SDR, generating fluorescent signals. **b**, Transcription kinetics for KL1-R1 and KL2-R2 in solution with free promoter oligonucleotide, monitored by plate reader ([NTP] : [Rx Reporter] : [$T_{KLx\text{-}Rx}$] : [p] = 100 : 10 : 1 : 1, 30 °C, 6 mM Mg^{2+}, 2.5 U/μL T7 RNAP, x = 1 or 2). Data are presented as mean ± SD from $N = 2$ independent experiments.

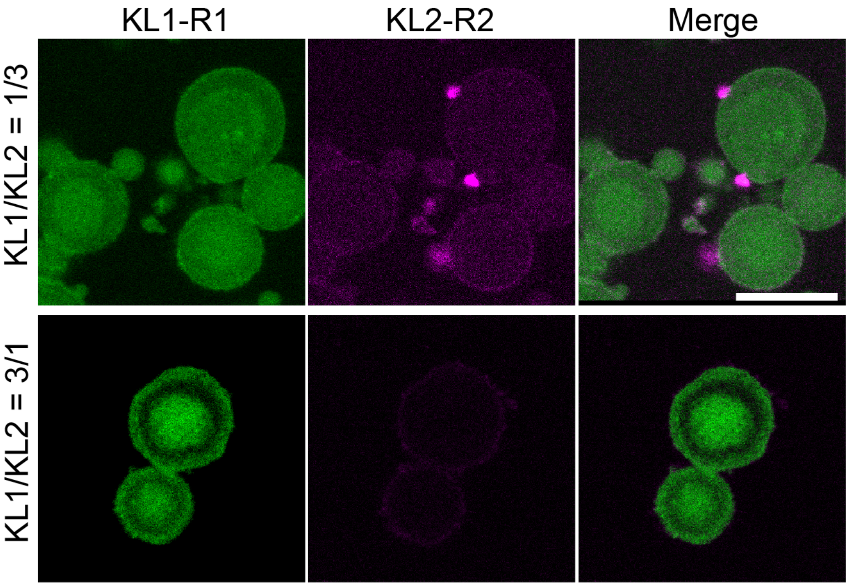

Supplementary Figure 14. Effect of asymmetric template concentrations for orthogonal KLs on the formation of condensates in PN.

Representative CLSM images of orthogonal KLs transcription in PN with 1/3 ratio (top) and 3/1 ratio (bottom) of $T_{KL1\text{-}R1}/T_{KL2\text{-}R2}$ at 30 Mg^{2+} ([NTP] : [R1*] : [R2*] : [$T_{KL1\text{-}R1}$] : [$T_{KL2\text{-}R2}$] : [p] = 3.6 : 1.8 : 1.8 : 0.75 : 0.25 : 1). Green channel: KL1-R1; Magenta channel: KL2-R2. Scale bar: 10 µm.

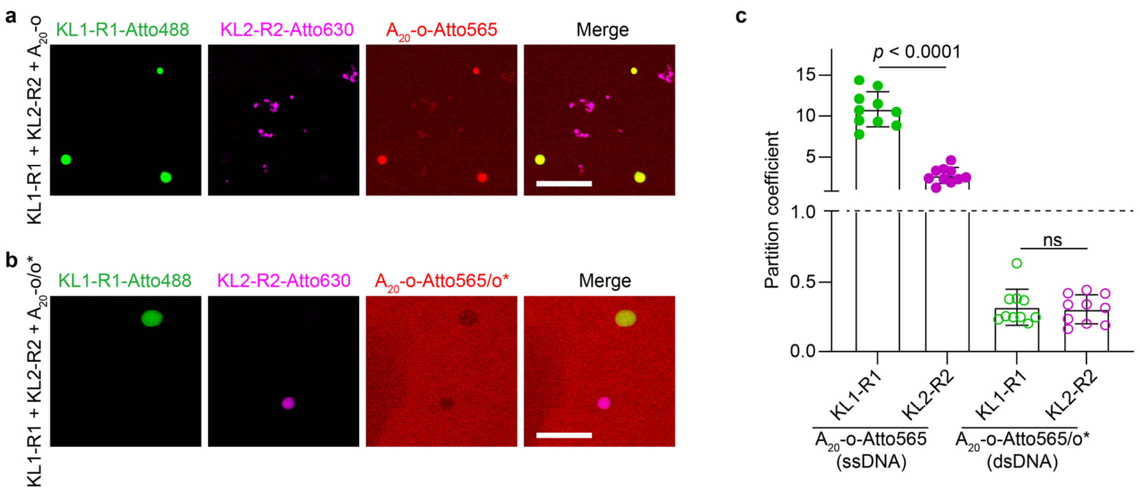

Supplementary Figure 15. Preferred partitioning of A_{20}-o ssDNA into KL1-R1 condensates and exclusion of A_{20}-o/o* dsDNA from both KL1-R1 and KL2-R2 condensates.

a, Representative CLSM images of mixed transcriptional KL1-R1 and KL2-R2 condensates in solution, with the addition of ssDNA A_{20}-o-Atto565 for 1 h. **b**, Representative CLSM images of mixed transcriptional KL1-R1 and KL2-R2 condensates in solution with the addition of dsDNA A_{20}-o-Atto565/o* for 1 h. In (**a**) and (**b**), both transcriptional condensates were initially prepared in separate reactions and subsequently mixed. ([NTP] : [$T_{KL1\text{-}R1}$ or $T_{KL2\text{-}R2}$] : [p] = 14.4 : 1 : 1, 30 °C, 30 mM Mg^{2+}, 18 h reaction). KL1-R1 and KL2-R2 transcripts are labeled with 1 mol% UTP-Atto488 or UTP-Atto630, respectively. Green channel: KL1-R1 condensate; Magenta channel: KL2-R2 condensate; Red channel: A_{20}-o-Atto565 ssDNA in (**a**) and A_{20}-o-Atto565/o* dsDNA in (**b**). **c**, Partition coefficient of ssDNA A_{20}-o-Atto565 and dsDNA A_{20}-o-Atto565/o* in different KL condensates, quantified by normalizing their intensity in KL condensates to their intensity in solution. $N = 10$ from 3 independent experiments. Data are presented as mean ± s.e.m. The statistical data were analyzed using a two-sided t-test. $p = 0.789$ for KL1-R1 *vs* KL2-R2 with addition of A_{20}-o-Atto565/o*. Scale bars: 10 μm (**a**, **b**).

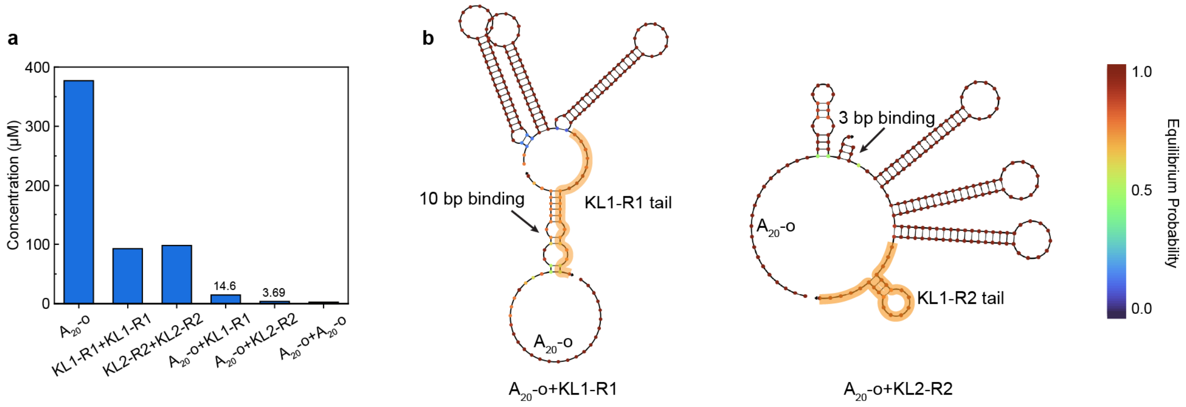

Supplementary Figure 16. Binding interaction between A_{20}-o, KL1-R1, and KL2-R2.

a, Simulated concentration distribution of complex sequences in a pool of A_{20}-o (400 μM), KL1-R1 (200 μM), and KL2-R2 (200 μM) at 30 °C based on rna06 model. Salt: 1 M Na^{+}. **b**, Predicted minimum of free energy (MFE) proxy structure at 30 °C of A_{20}-o + KL1-R1 and A_{20}-o + KL2-R2. Tails of KL1-R1 and KL2-R2 are highlighted in orange. The binding between A_{20}-o and KLs are indicated by arrows.

	Name	Sequence (5' → 3')	Purification	Modification	Supplier
Template and RCA	Tp(A20-p)	/Phosphate/ATA GTG AGT CGT ATT ATT TTT TTT TTT TTT TTT TTT ATC CCT	HPLC	5'-Phosphorylation	Biomers
	Tp(A20-o)	/Phosphate/TAC CTC AAT GCT TTT TTT TTT TTT TTT TTT TTG GGA GCA ACA A	HPLC	5'-Phosphorylation	Biomers
	Tp(T20-k)	/Phosphate/ATC CTC TAA AAT CAA AAA AAA AAA AAA AAA AAA GTA AAA CCA CAC G	HPLC	5'-Phosphorylation	Biomers
	ligation-p	TAA TAC GAC TCA CTA TAG GGA T	HPLC	None	Biomers
	ligation-o	GCA TTG AGG TAT TGT TGC TCC CA	HPLC	None	Biomers
	ligation-k	TTT TAG AGG ATC GTG TGG TTT T	HPLC	None	Biomers
	primer-p	TAA TAC GAC TCA CTA TAG GG*A*T	Desalting	Phosphorothioated twice (*)	IDT
	primer-o	GCA TTG AGG TAT TGT TGC TC C*C*A	Desalting	Phosphorothioated twice (*)	IDT
	primer-k	TTT TAG AGG ATC GTG TGG TT*T*T	Desalting	Phosphorothioated twice (*)	IDT
Label	Atto565-p*	/ATTO565/ATC CCT ATA GTG AGT CGT ATTA	HPLC	5'-Atto565	Biomers
	Atto647-o*	/ATTO647N/TGG GAG CAA CAA TAC CTC AAT GC	HPLC	5' Atto647N	Biomers
	Atto488-k*	/ATTO488/ AAA ACC ACA CGA TCC TCT AAA A	HPLC	5' Atto488	Biomers
	Atto647-k*	/ATTO647N/ AAA ACC ACA CGA TCC TCT AAA A	HPLC	5' Atto647N	Biomers
	Atto565-A20-o	/Atto565/ AAA AAA AAA AAA AAA AAA AA GCA TTG AGG TAT TGT TGC TCCCA	HPLC	5' Atto565	Biomers
	Atto647-A20-o	/Atto647N/ AAA AAA AAA AAA AAA AAA AA GCA TTG AGG TAT TGT TGC TCCCA	HPLC	5' Atto647N	Biomers

Supplementary Table 1. Oligomers for PN and labels, with their names, sequences, purification methods, modifications, and suppliers.

	Name	Sequence (5’ → 3’)	Purification	Modification	Supplier
Transcription	P (promoter)	TAA TAC GAC TCA CTA TAG GGA T	HPLC	None	Biomers
	Rep	/6-FAM/CTA CAT CCA CAT ACT A	HPLC	5’-6-FAM	Biomers
	Rep’	GTT AAT TAG TAT GTG GAT GTA G/BMN-Q1/	HPLC	3’-BMN-Q1	Biomers
	R1 Rep	/6-FAM/ATC TGA ACG AGT AAG G	HPLC	5’-6-FAM	Biomers
	R1 Rep’	TGG GGT CCT TAC TCG TTC AGA T/BMN-Q1/	HPLC	3’-BMN-Q1	Biomers
	R2 Rep	/6-FAM/GTG GCT TAT TTA CAG G	HPLC	5’-6-FAM	Biomers
	R2 Rep’	CTA ACG CCT GTA AAT AAG CCA C/BMN-Q1/	HPLC	3’-BMN-Q1	Biomers
	T_{Rep*} for plate reader	GTT AAT TAG TAT GTG GAT GTA GAT CCC TAT AGT GAG TCG TAT TA	HPLC	None	Biomers
	T_{X*} for CLSM	TTA GGA TAG ATA TAC GGG TTC ATC CCT ATA GTG AGT CGT ATT A	HPLC	None	Biomers
Kissing Loop	T_{KL1}	CACTCATAGCACTGTGCTTTCGCGATGCACAATGCTACGAGTGACGCGTACCTCAAAGGACTTTCGCGATGTCCTCTGAGGCACGCGAGCACACTAGAGCCGCTCTTTCGCGATGAGCGACTCTAATGTGCATCCCTATAGTGAGTCGTATTA	HPLC	None	IDT
	T_{KL1}’	GCACATTAGAGTCGCTCATCGCGAAAGAGCGGCTCTAGTGTGCTCGCGTGCCTCAGAGGACATCGCGAAAGTCCTTTGAGGTACGCGTCACTCGTAGCATTGTGCATCGCGAAAGCACAGTGCTATGAGTG	HPLC	None	IDT
	T_{KL1-R1}	TGGGGTCCTTACTCGTTCAGATGCACTCATAGCACTGTGCTTTCGCGATGCACAATGCTACGAGTGACGCGTACCTCAAAGGACTTTCGCGATGTCCTCTGAGGCACGCGAGCACACTAGAGCCGCTCTTTCGCGATGAGCGACTCTAATGTGCATCCCTATAGTGAGTCGTATTA	HPLC	None	IDT
	T_{KL1-R1}’	GCACATTAGAGTCGCTCATCGCGAAAGAGCGGCTCTAGTGTGCTCGCGTGCCTCAGAGGACATCGCGAAAGTCCTTTGAGGTACGCGTCACTCGTAGCATTGTGCATCGCGAAAGCACAGTGCTATGAGTGCATCTGAACGAGTAAGGACCCCA	HPLC	None	IDT
	T_{KL2-R2}	CTAACGCCTGTAAATAAGCCACCCACTCATAGCACTGTGCTTGTCGACTGCACAATGC	HPLC	None	IDT

	TACGAGTGACGCGTACCTCAAAGGACTTG TCGACTGTCCTCTGAGGCACGCGAGCACA CTAGAGCCGCTCTTGTCGACTGAGCGACT CTAATGTGCATCCCTATAGTGAGTCGTAT TA			
$T_{KL2\text{-}R2}$’	GCACATTAGAGTCGCTCAGTCGACAAGAG CGGCTCTAGTGTGCTCGCGTGCCTCAGAG GACAGTCGACAAGTCCTTTGAGGTACGCG TCACTCGTAGCATTGTGCAGTCGACAAGC ACAGTGCTATGAGTGGGTGGCTTATTTAC AGGCGTTAG	HPLC	None	IDT
Atto647-R1*	/ATTO647N/TCC TTA CTCG	HPLC	5’-Atto647N	Biomers
Atto488-R2*	/ATTO488/CGC CTG TAAA	HPLC	5’-Atto488	Biomers
$T_{KL1\text{-}BrA}$	GGAGCCCACACTCTACTCAACAGGCAACA TTTTTGTGCCTGGACCCGACCGTCTCCAAC ACTCATAGCACTGTGCTTTCGCGATGCAC AATGCTACGAGTGACGCGTACCTCAAAGG ACTTTCGCGATGTCCTCTGAGGCACGCGA GCACACTAGAGCCGCTCTTTCGCGATGAG CGACTCTAATGTGCATCCCTATAGTGAGT CGTATTA	HPLC	None	IDT
$T_{KL1\text{-}BrA}$’	GCACATTAGAGTCGCTCATCGCGAAAGAG CGGCTCTAGTGTGCTCGCGTGCCTCAGAG GACATCGCGAAAGTCCTTTGAGGTACGCG TCACTCGTAGCATTGTGCATCGCGAAAGC ACAGTGCTATGAGTGTTGGAGACGGTCGG GTCCAGGCACAAAAATGTTGCCTGTTGAG TAGAGTGTGGGCTCC	HPLC	None	IDT

Supplementary Table 2. Oligomers for kissing loop condensate transcription, with their names, sequences, purification methods, modifications, and suppliers.

* Represents a fully complementary sequence; ’ denotes a partially complementary sequence with a toehold.
