# Supplementary material for: Constructing synthetic nuclear architectures via transcriptional condensates in a DNA protonucleus
Source: Nat Commun. 2025 Sep 10;16:8254. doi: 10.1038/s41467-025-63445-8 (PMC12423304; doi:10.1038/s41467-025-63445-8)
Supplement: Supplementary file 3 — Description of Additional Supplementary Files [file 41467_2025_63445_MOESM3_ESM.pdf]

<u>Description of Additional Supplementary Files</u>

Supplementary Movie 1: LOCAL TRANSCRIPTION IN DNA PROTONUCLEUS (PN).

PN containing promoter sequence in its interior by phase separation of poly(A_{20}-p)$_n$ enables enrichment of DNA templates and spatial control of RNA transcription.

Supplementary Movie 2: CO-CONDENSATE OF TRANSCRIPTIONAL KL CONDENSATE AND DNA MATRIX IN PN.

The locally produced KL1 transcripts co-condense with the DNA matrix within PNs, which leads to a co-condensate depositing at the bottom of PNs.

Supplementary Movie 3: INVASION OF 'o*-Atto647' DISASSEMBLES CO-CONDENSATES IN PN.

By adding o* as an invader to hybridize with poly(A_{20}-o)$_n$ strand, the co-condensate experiences a continuous surface erosion, with a sharp interface defined by a bright ring of o*-Atto647. This process leads to the occupation of liberated poly(A_{20}-o)$_n$/o*-Atto647 within the entire space of PN, whereas the KL1 transcripts are squeezed to the PN periphery and eventually dissolve into solution.
